# Identification of an Au(I) N-Heterocyclic Carbene Compound as a Bactericidal Agent Against *Pseudomonas aeruginosa*

**DOI:** 10.3389/fchem.2022.895159

**Published:** 2022-04-28

**Authors:** Jinhui Wang, Xiaoshuai Sun, Yanxuan Xie, Yan Long, Huowen Chen, Xiaojun He, Taotao Zou, Zong-Wan Mao, Wei Xia

**Affiliations:** ^1^ School of Chemistry, Sun Yat-sen University, Guangzhou, China; ^2^ School of Ophthalmology and Optometry, School of Biomedical Engineering, Wenzhou Medical University, Wenzhou, China; ^3^ School of Pharmaceutical Sciences, Sun Yat-sen University, Guangzhou, China

**Keywords:** gram-negative, *Pseudomonas aeruginosa*, antibacterial agents, Au(I) N-heterocyclic carbene compound, bactericidal activity, bacterial resistance

## Abstract

The opportunistic pathogen *Pseudomonas aeruginosa* (*P. aeruginosa*) causes infections that are difficult to treat, which is due to the bacterial resistance to antibiotics. We herein identify a gold(I) N-heterocyclic carbene compound as a highly potent antibacterial agent towards *P. aeruginosa*. The compound significantly attenuates *P. aeruginosa* virulence and leads to low tendency to develop bacterial resistance. The antibacterial mechanism studies show that the compound abrogates bacterial membrane integrity, exhibiting a high bactericidal activity toward *P. aeruginosa*. The relatively low cytotoxic compound has excellent therapeutic effects on both the eukaryotic cell co-culture and murine wound infection experiments, suggesting its potential application as a bactericidal agent to combat *P. aeruginosa* infection.

## Introduction

*Pseudomonas aeruginosa* (*P. aeruginosa*) is a Gram-negative bacterium that exhibits extensive metabolic adaptability, enabling it to thrive in an extensive range of niches. It perfectly evolved to be a successful opportunistic pathogen, causing wide range of acute, and chronic infections (Mah et al., 2003; Wu et al., 2011; Lund-Palau and Turnbull, 2016; Lee and Yoon, 2017). For example, patients with cystic fibrosis (CF) or immunodeficiency are peculiarly susceptible to the *P. aeruginosa* infections (Shteinberg et al., 2021). *P. aeruginosa* has inherent resistance to a series of antibiotics through various mechanisms, such as reducing outer membrane permeability, expression of efflux systems that pump antibiotics out of the cell (Jürg and Paolo, 2015; Verchère et al., 2015). Moreover, *P. aeruginosa* can acquire drug resistance by either horizontal transfer of resistance genes or mutational changes (Botelho et al., 2019; Hernando-Amado et al., 2020). Therefore, the infections caused by this organism are notoriously difficult to treat. Thus, there is an urgent need for the development of new antimicrobial reagents with distinct mechanism of action to combat *P. aeruginosa* infection (Martin et al., 2020; Mitcheltree et al., 2021).

Metal-containing compounds have exerted a seminal role in medicinal chemistry. A series of metal complexes are currently in clinical use for the treatment of bacterial infections (Chernousova and Epple, 2013; Fischbach and Malfertheiner, 2018). These metal compounds usually have unique mechanism of action, such as ligand exchange or release, reactive oxygen species (ROS) generation (Liao et al., 2017a; He et al., 2017; Han et al., 2018). Particularly, these metal compounds have multi-target properties to inhibit bacterial growth and thereby reducing the development of drug resistance of bacteria when compared with the conventional antibiotics (Gasser and Metzler-Nolte, 2012; Frei et al., 2020). Recent studies on drug repositioning led to the discovery of auranofin, a drug initially approved as an anti-rheumatic agent, exhibiting potent antibacterial activity (Harbut et al., 2015; Thangamani et al., 2016; Lu et al., 2020; Sun et al., 2020). Auranofin is active against a broad-spectrum of Gram-positive bacteria via inhibition of the thioredoxin reductase, thereby sensitizing the bacteria to oxidative stress (Harbut et al., 2015). Although auranofin showed promise for treatment of infectious diseases caused by Gram-positive bacteria, the metal compound lacked bactericidal activity toward Gram-negative bacteria, impeding its practical application.

Herein, we reported that a mononuclear gold compound, NHC^Me^-Au-Cl (NHC = N-heterocyclic carbene), named as Au1, exhibited high bactericidal activities to both Gram-negative and Gram-positive bacteria. Similar to auranofin, Au1 could significantly inhibit the virulence factors of *P. aeruginosa*. Importantly, repeated use of the gold compound does not lead to bacterial drug resistance. Moreover, the treatment of Au1 led to severe bacterial membrane crumpling, and thereby disrupted the membrane integrity of *P. aeruginosa*. Further transcriptomic and proteomic analysis showed that Au1 and auranofin have different mechanism of action. Au1 substantially disturbed the trehalose biosynthesis and riboflavin metabolism pathways in *P. aeruginosa*. The anti-bacterial activities of **Au1** were further verified *in vivo* using murine skin infection experiments. Our studies revealed the potential of the **Au1** compound as a novel bactericidal reagent to combat the *P. aeruginosa* infections.

## Materials and Methods

### Gold(I) Compound Preparation

Au4 and Au9 (auranofin) are purchased from Sigma-Aldrich. Au1–Au3 (Hickey et al., 2008; Long et al., 2021), four dinuclear gold(I)-phosphine compounds Au5–Au8 (Che et al., 1990; Fu et al., 1999; Igashira-Kamiyama et al., 2013; Zou et al., 2014) and two auranofin derivatives Au10–Au11 (Baker et al., 2005) were synthesized and characterized as previously described.

### Bacterial Strains and Growth Condition

*Pseudomonas aeruginosa* strain PAO1 (*P. aeruginosa*) was grown in M9 minimal medium (1.0 g/L NH_4_Cl, 0.5 g/L NaCl, 6.0 g/L Na_2_HPO_4_, 3.0 g/L KH_2_PO_4_) supplemented with 1.0 mM MgSO_4_, 1.0 mM ZnSO_4_, 0.2 mM CaCl_2_, 0.4% (w/v) glucose and 0.2% (w/v) casein hydrolysate. *Gram*-negative *Escherichia coli* strain K-12 (*E. coli*) and *Burkholderia cepacia J2315* (*B. cepacia*) were cultured in Luria-Bertani (LB) medium (5 g/L yeast extract, 10 g/L tryptone, 10 g/L sodium chloride). The *Gram*-positive *Staphylococcus aureus* strain Newman (*S. aureus*) was grown in Tryptic Soy Broth (TSB) medium (17 g/L Tryptone, 3 g/L Phytone, 5 g/L sodium chloride, 2.5 g/L dipotassium phosphate and glucose 2.5 g/L).

### Antimicrobial Activity

The minimal inhibitory concentration (MIC) values of the gallium compounds were determined using a standard broth dilution method as described previously (Wiegand et al., 2008). In brief, the compounds were two-fold serially diluted to concentrations from 400 to 1.56 μM. Approximately 50 μL of the compound solution was added to a 96-well microtiter plate and mixed with 50 μL of bacteria culture in log phase (10^6^ CFU/ml). The bacterial culture was further incubated at 37°C for 22–24 h. The OD_600_ values for each well was recorded on a microplate reader. The MICs value was defined as the lowest compound concentration that inhibited 95% of the bacterial growth.

### Cytotoxicity in Mammalian Cell

The cytotoxicity of gold compounds to mammalian cells was measured using the 3-(4, 5-dimethylthiazol-2-yl)-2, 5-diphenyltetrazolium bromide (MTT) assay. Human hepatocyte L-O2 cell, and lung carcinoma A549 cell were cultured in Corning 96 well microplates (10^4^ cells per well) at 37°C for 18 h. Then the cells were incubated with gradient concentrations of gold compounds at 37°C for another 48 h. After the treatment, cell culture media were removed and the plates were wash with PBS for three times. And 90 μL fresh media and 10 μL 5 mg/ml MTT solution were added to each well and further incubated at 37°C for 4 h. Subsequently, the media were removed from the wells and 150 μL DMSO was added into each well to dissolve the formazan crystals. The absorbance at 490 nm of each well was measured with a micro-plate reader. The experiments were carried out in triplicates.

### Measurement of Au Contents

The amount of gold compound uptake by *P. aeruginosa* was quantified by ICP-MS. *P. aeruginosa* overnight culture was diluted to OD_600_ of 0.1 in the presence of 15 μM of **Au1** or auranofin. Approximately 5 ml aliquot of bacterial culture was removed and collected every 2 h, up to 8 h. After washing with cold PBS supplemented with 5 mM EDTA (Sigma-Aldrich), cell pellets were digested with 70% HNO_3_ at 60°C overnight. Samples were then diluted with ddH_2_O to a final concentration of ca. 5% HNO_3_ and analyzed by ICP-MS (iCAP RQ, Thermo Fisher).

### Drug Resistance Development Assay

Development of resistance to the Au1 compound and the antibiotics (ciprofloxacin and tobramycin) were assessed as described by Habets and Brockhurst (2012). The MIC assay was performed on the first day as described above. On the next day, the MIC was recorded as the minimal concentration that completely inhibited growth. Thereafter, a new passage MIC measurement assay was prepared using diluted bacterial cells grown at 0.5-fold MIC suspension. This was repeated for 14 passages and the MIC values for Au1, ciprofloxacin and tobramycin were recorded for each passage.

### Quantification of Bacterial Virulence Factors

To quantify the secreted virulence factors, overnight *P. aeruginosa* PAO1 was diluted in fresh M9 medium and cultured to a final OD_600_ value of 0.15. Then Au1 or auranofin were added into culture with indicated concentrations. After 24 h incubation at 37°C, the bacterial supernatants were collected by centrifugation at 4,000 g for 10 min at 4°C and filtered using a 0.22 μm syringe filter (Millipore). The secreted virulence factors in the supernatant were quantified by the methods described below.

For LasA protease detection, 5 μL of filtered supernatant was mixed with 1 ml of 0.5% azocasein (Sigma-Aldrich) in reaction buffer (50 mM Tris-HCl, 0.5 mM CaCl_2_, pH 7.5). The mixture was incubated at 37°C for 15 min. After reaction, 500 μL of 10% trichloroacetic (TCI Shanghai) acid was added and incubated for 30 min at room temperature. After centrifugation at 10,000 g for 20 min, the 440 nm absorbance of the collected supernatant was measured to quantify the secreted LasA protease.

The LasB elastase was examined by Elastin-Congo red assay (Morihara et al., 1965). In brief, 700 μL of bacterial supernatant was incubated with 700 μL of 10 mg/ml Elastin-Congo (Sigma-Aldrich) red elastase substrate in reaction buffer (100 mM Tris-HCl, 1 mM CaCl_2_, pH 7.5) at 37°C for 3 h with shaking. Then 0.2 ml EDTA was added to a final concentration of 10 mM to stop the cleavage reaction. Insoluble pellets were removed by centrifugation and the absorbance of the supernatant at 495 nm was measured.

The bacterial secreted pyocyanin was quantified as described previously (Essar et al., 1990). Approximately 10 ml bacterial supernatant medium was collected by centrifugation. Subsequently, 3 ml chloroform was added into medium to extract the pyocyanin. The pyocyanin was re-extracted into 1 ml of 0.2 M HCl to give a pink to deep red solution after approximately 30 min. The pyocyanin concentration was determined by measurement the absorbance at 520 nm.

For rhamnolipid measurement, rhamnolipid was extracted from the supernatant with a mixture of chloroform and methanol (v:v = 2:1). The organic solvent phase was then evaporated to dryness and 100 μL methanol was added to dissolve the extracted rhamnolipid. Then 200 μL of pre-cooled anthrone solution (dissolving 2 g of anthrone in 1 L of concentrated H_2_SO_4_) was added into 20 μL extracted rhamnolipid sample and heat at 80°C for 30min. The rhamnolipid concentration was determined by measurement the absorbance at 630 nm.

### *P. aeruginosa* Biofilm Inhibition

*P. aeruginosa* was cultured in 24-well microtiter plate (Corning, NY, United States) for static biofilm formation. For biofilm inhibition assay, approximately 1 ml of bacterial overnight culture were diluted in fresh M9 medium with a final OD_600_ value of 0.33 and transferred into each well of microtiter plate in the absence or presence of sub-lethal concentration of Au1 or auranofin (2.5, 10, and 15 μM). After static incubation at 37°C for 48 h, weakly adherent planktonic cells were removed from the microtiter plate by washing with phosphate buffered saline (PBS) for three times gently. The adherent biofilms were dried and stained with 0.1% (w/v) crystal violet for 15 min. Excess dye was removed by rinsing the plate three times with PBS. Crystal violet was solubilized from the stained biofilms by adding 30% acetic acid and the biofilms were quantified by measuring the 595 nm absorbance. Each experiment was performed in triplicates.

### Field Emission Scanning Electron Microscopy

Images of *P. aeruginosa* were recorded on a JSM-6330F (JEOL) field emission scanning electron microscopy as previously reported (Zhao et al., 2021). *P. aeruginosa* was cultured in M9 medium until OD_600_ reached 0.6–0.8. Au1 or auranofin was added to medium to a final concentration of 50 µM and further incubated at 37°C for 1 h with shaking. After washing twice with PBS buffer, the samples were subsequently fixed using 2.5% glutaraldehyde in PBS buffer at 4°C overnight. After rinsing twice with PBS buffer, the samples were subsequently exposed to ethanol dehydration series of 30, 50, 70, 90% for 10 min and 100% for 30 min. All the samples were finally air-dried in a vacuum desiccator and sputter-coated with palladium-gold thin film before data collection.

### Atomic Force Microscopy

For AFM imaging of bacterial cells, *P. aeruginosa* was cultured in M9 medium until OD_600_ reached 0.6–0.8. Au1 or auranofin was added to medium to a final concentration of 50 µM and further incubated at 37°C for 1 h. Diluted bacterial suspension was added on a dry glass slide that is pre-treated with poly-L-Lysine. After immobilization for 1 h, excess liquid was removed and the glass slide was wash with PBS gently. Finally, dry glass slide was subjected to a Dimension FastScan^®^ atomic force microscope (Bruker) with tapping mode.

### Bacterial Membrane Permeability Assay

Overnight *P. aeruginosa* culture was diluted 1:100 with fresh M9 medium and grown to early-mid exponential phase (OD_600_ = 0.4–0.6). The culture was then diluted 1:10 into PBS and treated with the 50 μM concentration of Au1 or auranofin for 30 min. Then the bacteria were incubated with propidium iodide (PI), a dye that is excluded from cells with impermeable membrane. The CytoFLEX flow cytometry (Beckman Coulter) was used to measure the bacterial fluorescent intensities and the data was analyzed using CytExpert 2.4 software. For the time-course detection of the bacterial permeability induced by Au1 or auranofin, *P. aeruginosa* in early-mid exponential phase (OD_600_ = 0.2) was incubated with PI dye on a 96-well plate. **Au1** or auranofin was added to a final concentration of 50 μM. The fluorescence signal (561 nm excitation, 590 nm emission) and OD_600_ value were recorded on Cytation5 microplate reader. Each experiment was performed in triplicates.

### Transcriptomic Assay and Data Processing

*P. aeruginosa* cultures were grown to early log phase (OD_600_ = 0.3) and Au1 (or auranofin) compound was added to a final concentration of 15 μM and the bacteria were further cultured for 4 h at 37°C. And total RNA was isolated from the cultured *P. aeruginosa* cells using SV total RNA isolation kit (Promega) according to the manufacturer’s instructions. And RNA was quantified by NanoDrop spectrophotometer. The quality of the isolated RNA was examined by Agilent 2,100 Bioanalyzer (Agilent). Paired-end, strand-specific RNA-seq was performed using the dUTP method (Levin et al., 2010). The ribosomal RNAs were removed with Ribo-Zero rRNA Removal Kit (Illumina). Subtracted RNAs were fragmented and reverse-transcribed. The sequencing library was loaded on an Illumina HiSeq instrument according to manufacturer’s instruction. RNA-seq experiments were performed in triplicates.

### Co-Culture Experiment

Human alveolar basal epithelial A549 cells were cultured in 1,640 medium. And Au1 was added into medium to different final concentrations (10, 20, and 30 μM). *P. aeruginosa* overnight culture was diluted 10 times by 1,640 medium and incubated at 37°C for 2 h. Subsequently, the adapted *P. aeruginosa* bacterium was added into A549 cell culture to a final concentration of 10^6^ CFU/ml and the culture was further incubated at 37°C for 2 h. The morphology of the cells was recorded on a Nikon eclipse TS100 inverted microscopy.

### Mouse Skin Infection

Six-week-old female BALB/c mice were used for animal experiments to evaluate the healing effect of infected wounds *in vivo*. Briefly, mouse backs were cut to generate a circular wound (diameter 0.6 cm) with a hole punch and the wound was infiltrated using a medical cotton swab with *P. aureginosa* (overnight culture, OD_600_ = 0.3). The mice were randomly divided into three groups, including PBS, auranofin, and Au-1 groups (n = 5 in each group). The drugs were dissolved in DMSO and diluted with glycerol to a final concentration of 50 μM. After the bacterial fluid was dry, about 20 μL of the drug was added to the wound. Body weights and wound photography was recorded on days 0, 1, 3, 5, and 8. And on the 8th day, the mice were euthanized by chloral hydrate, the infectious tissues in all groups were collected and cultured into the LB plate for colony counting. The skin tissues were also fixed by paraformaldehyde solution (4%) for H&E, Gram, and Masson’s trichrome staining.

## Results and Discussion

### Identification of Bactericidal Gold(I) Compounds

**FIGURE 1:**
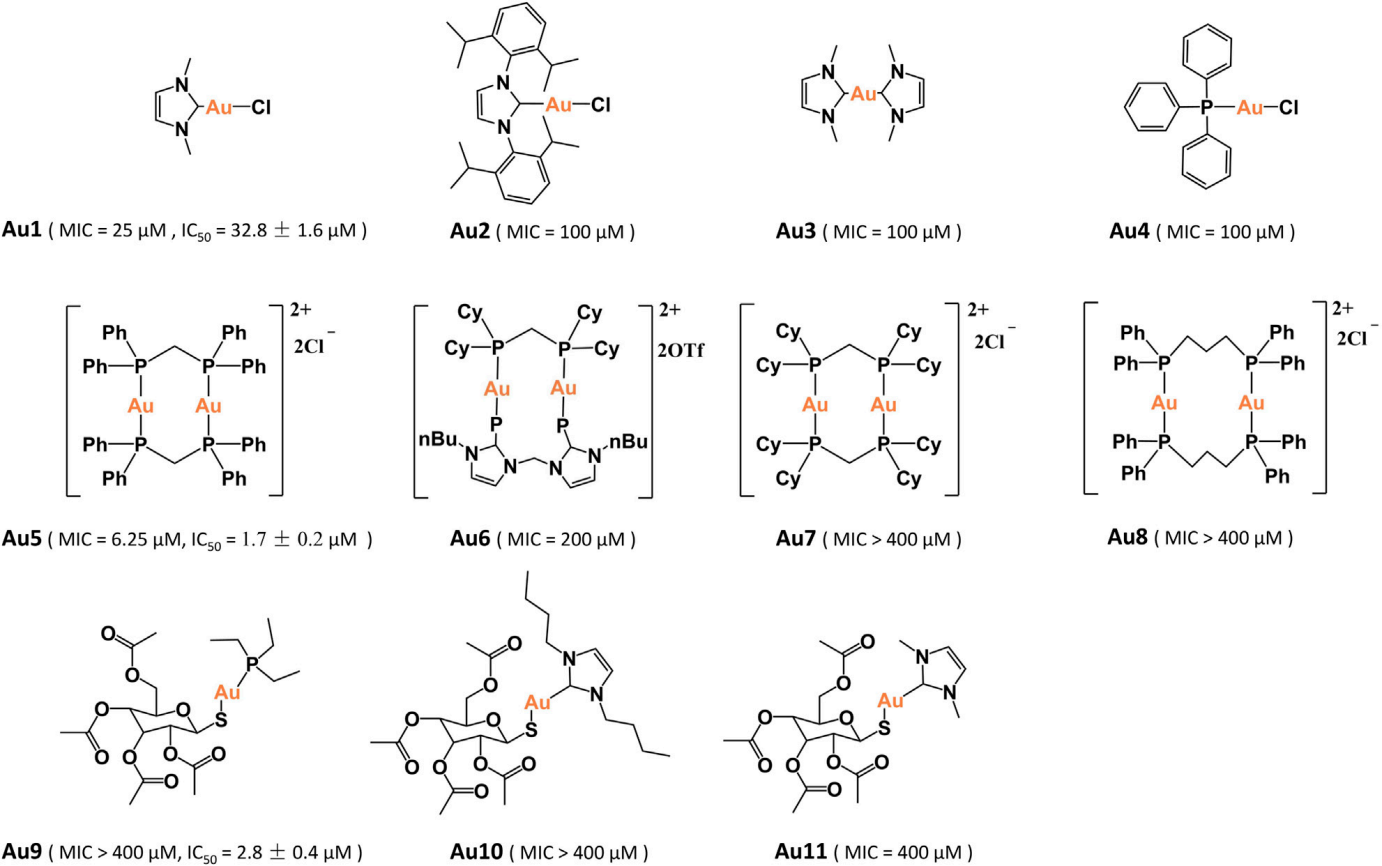
Chemical structure of the gold(I) compounds.

In total, eleven gold(I) compounds were used to examine the anti-bacterial activities against *P. aeruginosa* PAO1 strain (Figure 1). These compounds included four mononuclear gold(I) compounds Au1–Au4, four dinuclear gold(I)-phosphine compounds Au5–Au8, auranofin (Au9) and two auranofin derivatives Au10–Au11. The minimal inhibitory concentrations (MIC) of these compounds were shown in Table 1. All the mononuclear gold(I) carbene compounds exhibited substantial bactericidal activities toward *P. aeruginosa*, among which Au1 had the lowest MIC value of 25 μM. Au2 that contains a larger NHC^IPr^ carbene ligand, had a higher MIC value (100 μM), indicative of the essential role of the NHC^Me^ ligand for the bactericidal activity of gold compound. Unexpectedly, the four dinuclear gold(I)-phosphine compounds exhibited significantly different bactericidal activities. Au5 is highly toxic to the bacteria with a MIC value of 6.25 μM. In contrast, Au6–Au8 exhibited much weaker or no bactericidal activities. The high bactericidal activity of lipophilic Au5 is probably due to its enhanced cell permeability. However, increasing lipophilicity of Au6 and Au7 led to poor water solubility, which may partially explain their low antibacterial activities. Whereas, the instability of Au8 in solution might contribute to its low activity (Berners-Price and Sadler, 1986). In addition, the auranofin and two derivatives had no bactericidal activities toward *P. aeruginosa* with the MIC values higher than 400 μM. In line with the determined MIC values, bacterial growth curves showed that the *P. aeruginosa* growth was completely inhibited in the presence of 20 μM Au1 or 3 μM Au5. In contrast, as high as 25 μM aranofin exhibited little impact on the bacterial growth (Supplementary Figure S1). Therefore, the mononuclear Au1 and dinuclear Au5 are the two most potent bactericidal gold compounds.

**TABLE 1:** The minimal inhibitory concentrations (MIC) of Au1–Au11 towards *P. aeruginosa*.

Compound	MIC (μM)
Au1	25
Au2	100
Au3	100
Au4	100
Au5	6.25
Au6	200
Au7	>400
Au8	>400
Au9	>400
Au10	>400
Au11	400

Subsequently, the mammalian cell cytotoxicity of Au1, Au5, and auranofin were examined using A549 and LO2 cell lines. The MTT assays showed that Au5 and auranofin had high cytotoxicity to A549 cell, with the IC_50_ values of 1.7 ± 0.2 and 2.8 ± 0.4 μM, respectively. While the cytotoxicity of Au1 is much lower with an IC_50_ value of 32.8 ± 1.6 μM. Similar results were also obtained for the LO2 cells (Supplementary Figure S2). Given the high bactericidal activity and relatively low cytotoxicity of Au1, we then focused on Au1 and characterized its antibacterial activity.

### Characterization of the Antibacterial Activity of Au1

Although **Au1** can effectively eliminate *P. aeruginosa,* the N-heterocyclic carbine ligand had no effect on bacterial growth even at 50 μM, indicating that the intact compound structure is required for the high bactericidal activity (Supplementary Figure S3). We subsequently examined the antibacterial activities of Au1 against other common bacterial species, including *Staphylococcus aureus Newman*, *Burkholderia cepacia J2315* and *Escherichia coli MG16552*. The sensitivity of these pathogens to Au1 compound and four antibiotics were investigated (Supplementary Table S1). The results showed that Au1 exhibited broad-spectrum antibacterial activities against all the four bacteria species, which is comparable to the ciprofloxacin.

**FIGURE 2:**
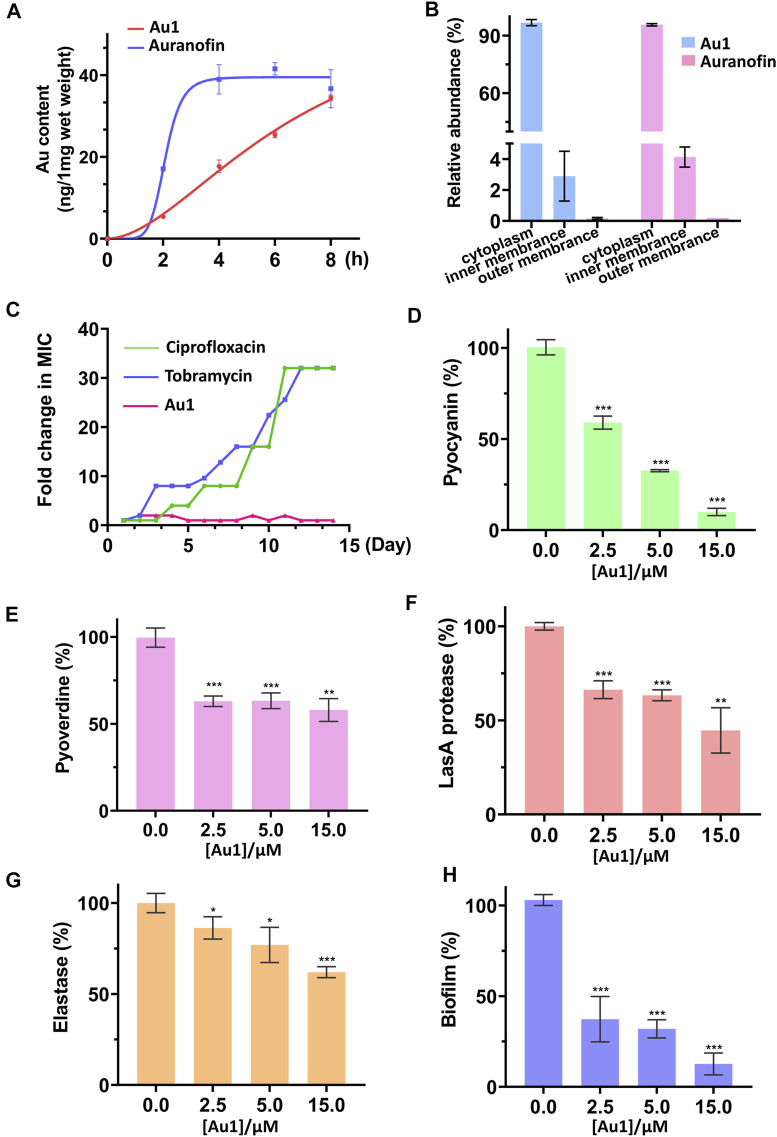
**(A)** Kinetics of Au1 and auranofin uptake by *P. aeruginosa*. **(B)** Subcellular distribution of Au contents in bacteria after Au1 or auranofin treatment **(C)** Bacterial resistance studies of Au1, ciprofloxacin and tobramycin clinical against *P. aeruginosa*. The inhibition of bacterial pyocyanin **(D)**, pyoverdine **(E)**, LasA protease **(F)**, elastase **(G)** and biofilm formation **(H)** by Au1 compound with different concentrations. All experiments are performed in triplicated and data are presented as mean ± sd. * means *p* < 0.05, ** means *p* < 0.01, *** means *p* < 0.001.

Previous studies indicated that the low bactericidal activity of auranofin was due to the two-membrane barrier of Gram-negative bacteria (Breijyeh et al., 2020; Wei Tan et al., 2020). Therefore, we set out to examine the bacterial uptake rates of Au1 and auranofin using inductively coupled plasma mass spectrometry (ICP-MS). Unexpectedly, both the auranofin and Au1 could be efficiently uptaken by *P. aeruginosa* with a final cellular concentration of approximately 40 ng/mg bacterial wet weight. Typically, auranofin even had a faster uptake rate compared to Au1 (Figure 2A). Further subcellular distribution analysis indicated that vast majority of the two compounds were located in the cytoplasm and only a tiny fraction remained on the bacterial membrane (Figure 2B). The results showed that the high bactericidal activity of Au1 was not associated with its uptake. Moreover, a recent study demonstrated that auranofin could synergize with antibiotics on killing pathogenic bacteria (Sun et al., 2020). Encouragingly, similar synergistic effects were also observed between Au1 and two antibiotics used in clinic (tobramycin and colistin), suggesting that the Au1 could also be used as an adjuvant for antibiotic therapy (Supplementary Figure S4).

Since antimicrobial resistance is a major concern for the development of antibacterial regents, resistance development experiments were then employed to examine the bacterial resistance to Au1. And two antibiotics ciprofloxacin and tobramycin were used as control. As shown in Figure 2C, development of resistance to Au1 was not observed after a serial passage of *P. aeruginosa* in the presence of sub-lethal concentrations of Au-1 over 14 days. In contrast, the MIC values of ciprofloxacin and tobramycin both increased by 30 times over 14 days. The result indicates that continuous treatment of Au1 would not lead to the development of resistance by *P. aeruginosa*.

*P. aeruginosa* expresses numerous virulence factors that not only accelerate bacterial colonization but also elude immune clearance by the host. Previous studies showed that auranofin effectively attenuated the virulence factors expression of *P. aeruginosa* (Wei Tan et al., 2020). Given the high bactericidal activity of Au1, we subsequently examined the effects of Au1 on the bacterial virulence factors expression. *P. aeruginosa* bacterial culture was incubated with gradient concentrations of **Au1** that are sub-lethal to the bacteria. The inhibitory effects of Au1 on the pyocyanin, pyoverdine, LasA protease, and LasB elastase of *P. aeruginosa* were shown in Figures 2D–G. Pyocyanin is a secondary metabolite secreted by *P. aeruginosa* is able to target a wide range of cellular pathways and therefore can kill mammalian lung cells upon bacterial infection (Lau et al., 2004). The secreted pyocyanin was significantly reduced after incubation with Au1. Typically, the pyocyanin secretion was almost completely inhibited in the presence of 15 μM Au1. Similarly, the secretion of LasA protease, elastase and pyoverdine siderophore were also decreased by 54, 38, and 50% respectively after incubation with 15 μM Au1. Moreover, rhamnolipid is a unique class of glycolipid produced by *P. aeruginosa*. Previous study showed that rhamnolipid is the only identified virulence factor that was associated with the deterioration of the patients to ventilator-associated pneumonia in hospital (Wagener et al., 2021). Both the auranofin and Au1 (15 μM**)** could significantly suppress the synthesis of rhamnolipid (Supplementary Figure S5). Since the formation of rigorous biofilm is critical to the persistent infection of *P. aeruginosa,* we also examined whether Au1 has an effect on the formation of biofilms. Indeed, Au1 is sufficient to inhibit biofilm formation as low as 2.5 μM (Figure 2H). Collectively, all these results showed that the Au1 compound is a highly potent inhibitor to attenuate the virulence factor expression in *P. aeruginosa*.

### Au1 Disrupts the Bacterial Membrane Integrity

**FIGURE 3:**
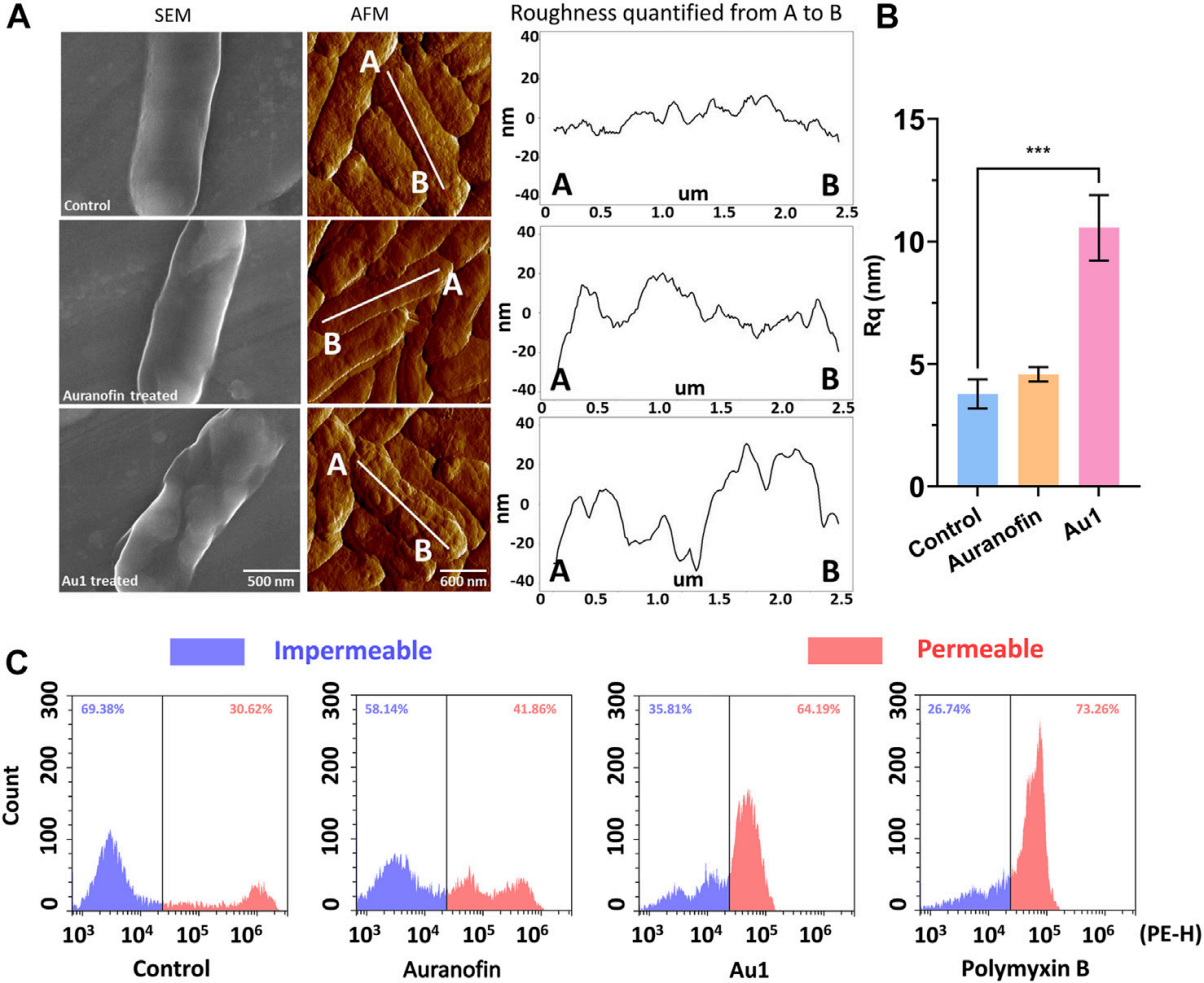
**(A)** FE-SEM and AFM analysis of the morphology of *P. aeruginosa* after incubation with Au1 or auranofin. **(B)** Quantification of the root-mean-square roughness (R_q_) of the bacterial surface. All experiments are performed in triplicated and data are presented as mean ± sd. *** means *p* < 0.001. **(C)** Flow cytometry analysis of the bacterial membrane permeability after incubation with auranofin, Au1 and polymyxin B. Blue colour represents the counted cell with impermeable membrane, and red colour represents the cell with permeable membrane.

The unexpected bactericidal activity of Au1 prompted us to further investigate its mechanism of action. We employed the field emission scanning electron microscope (FE-SEM) and atomic force microscopy (AFM) to visualize the morphological changes of *P. aeruginosa*. FE-SEM images showed that *P. aeruginosa* exhibited wrinkling and collapsed structures after the treatment of Au1, resulting in a rough bacterial surface with clear bumps and valleys. In contrast, no significant changes were observed in the auranofin-treated group (Figure 3A; Supplementary Figure S6). To further characterize the change of bacterial membrane after treatment, AFM was employed to measure the bacterial surface roughness (Figure 3A; Supplementary Figure S7). In consistent with the FE-SEM results, the root-mean-square roughness (R_q_) of the bacterial surface after Au1 treatment was determined to be 10.5 ± 1.5 nm, which is significantly larger than that in the control (4.5 ± 0.3 nm) and auranofin treated groups (3.8 ± 0.7 nm) (Figure 3B).

The significant change of bacterial surface roughness after Au1 treatment implied that the gold compound might damage the physiological function of bacterial membrane. Therefore, we further examined the bacterial permeability after treatment with Au1 or auranofin. In brief, log-phase *P. aeruginosa* was incubated with 50 μM auranofin, Au1 or 5 μM polymyxin B for 30 min followed by staining with propidium iodide (PI) dye to monitor the bacterial permeability change. Since the PI is membrane-impermeable and is generally excluded from viable cells, cells with increased permeability will produce a higher fluorescence signal than control cells. As shown in Figure 3C, auranofin treatment did not significantly affect the bacterial fluorescence signal when compared with the control group. Whereas, the treatment of polymyxin B, an antibiotic that targets and destabilizes bacterial outer membrane, remarkably increased the bacterial fluorescence signals. Similar fluorescence incensement was also observed in Au1 treated *P. aeruginosa*, indicative of enhanced bacterial membrane permeability. Moreover, we further investigated the kinetics of the bacterial membrane permeation caused by Au1 compound. *P. aeruginosa* bacteria were incubated in M9 medium supplemented with PI dye and different concentrations of Au1 or auranofin were added into the medium. Time-course of the fluorescence signals (561 nm ex, 590 nm em) were monitored on a microplate reader. The results showed that the initiation of bacterial membrane rupture occurred after 1.5 h when incubated with 50 μM Au1 at 37°C (Supplementary Figure S8). Collectedly, all the results clearly indicated that Au1 could disrupt the bacterial membrane integrity, and thereby leading to bacterial death.

### Transcriptomic Analysis

**FIGURE 4:**
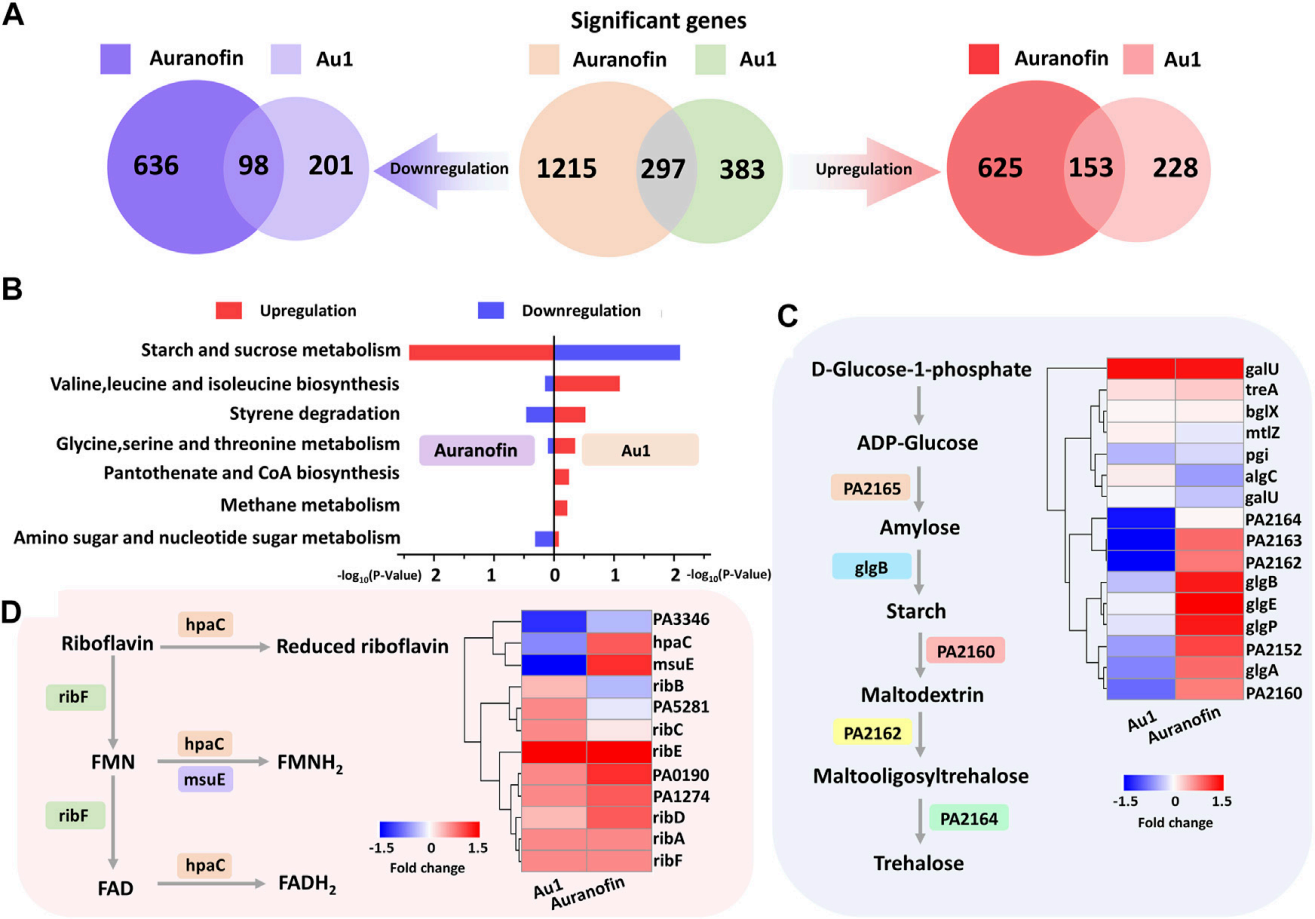
**(A)** Venn diagrams showing the number of up- and down-regulated genes in the Au1 and auarnofin treated groups compared to the control group. **(B)** KEGG analysis reveal enrichment pathways that completely opposites under the treatment of Au1 and Auranofin (left panel represents auranofin-treated group, right panel represents Au1-treated group). **(C)** Starch and sucrose metabolism pathway and the heat map for relative genes of *P. aeruginosa* in the Au1 and auranofin treated groups. **(D)** Riboflavin biosynthesis pathway and the heat map for relative genes of *P. aeruginosa* in the Au1 and auranofin treated groups.

Subsequently, we further investigated the antibacterial mechanism of Au1 on *P. aeruginosa* at the transcriptome levels using auranofin as a control. For transcriptomic analysis, log-phase *P. aeruginosa* was treated with 15 μM Au1 or auranofin for 4 h, from which good quality RNA were extracted, indicating that the bacteria were not severely damaged. Compared to the control group, the transcriptional levels of 680 genes were significantly altered by Au1 treatment, among which 381 and 299 genes were up- and down-regulated, respectively. Whereas, auranofin caused larger fluctuations in gene transcriptional levels than Au1 did, leading to a change of 1,512 gene, including 778 genes up-regulated, and 734 genes down-regulated (Figure 4A; Supplementary Figure S9). The significantly altered genes in Au1 and auranofin treated groups were analyzed according to the Kyoto Encyclopedia of Genes and Genomes (KEGG) database. Several KEGG pathways were identified in both Au1 and auranofin treated groups, such as ABC transporters, quorum sensing and starch and sucrose metabolism (Supplementary Figure S10). Unexpectedly, further analysis revealed different changes of the genes involved in the starch and sucrose metabolism pathway, i.e., the transcriptional levels of these genes were prominently up-regulated in auranofin-treated group, but down-regulated in Au1-treated group (Figure 4B). The starch and sucrose metabolism pathway is mainly responsible for the biosynthesis of glycogen and trehalose (Figure 4C). Since trehalose is essential for bacterial membranes stabilization under adverse stress conditions, this may at least partially explain why Au1 could severely damage bacterial membrane (Cross et al., 2018; Kim et al., 2021).

Since Au1 and auranofin exhibited different bactericidal activities, implying the two compounds should have distinct mechanism of actions. Transcriptomic analysis revealed that totally 73 KEGG pathways were both significantly perturbed by Au1 and auranofin, among which 44 pathways were affected by auranofin alone and 6 pathways were affected by Au1 alone (Supplementary Figure S11). Intriguingly, among the six pathways that are solely affected by Au1, four are related to the degradation of benzene homologues, one is related to sphingolipid metabolism and the last one is related to riboflavin metabolism. Riboflavin has an essential role in oxidative metabolism for all organisms. Particularly, it is also involved in quorum sensing signaling processes in bacteria (Li et al., 2021). The Au1 treatment significantly down-regulated the transcriptional level of *hpaC* and *msuE* genes, which encode the reductase involved in flavin mononucleotide (FMN) and flavin adenine dinucleotide (FAD) biosynthesis (Figure 4D). The results implied that the abrogation of the essential riboflavin metabolism pathway may contribute to the high bactericidal activity of Au1.

### Mammalian and Bacterial Cells Co-Culture Experiment

**FIGURE 5:**
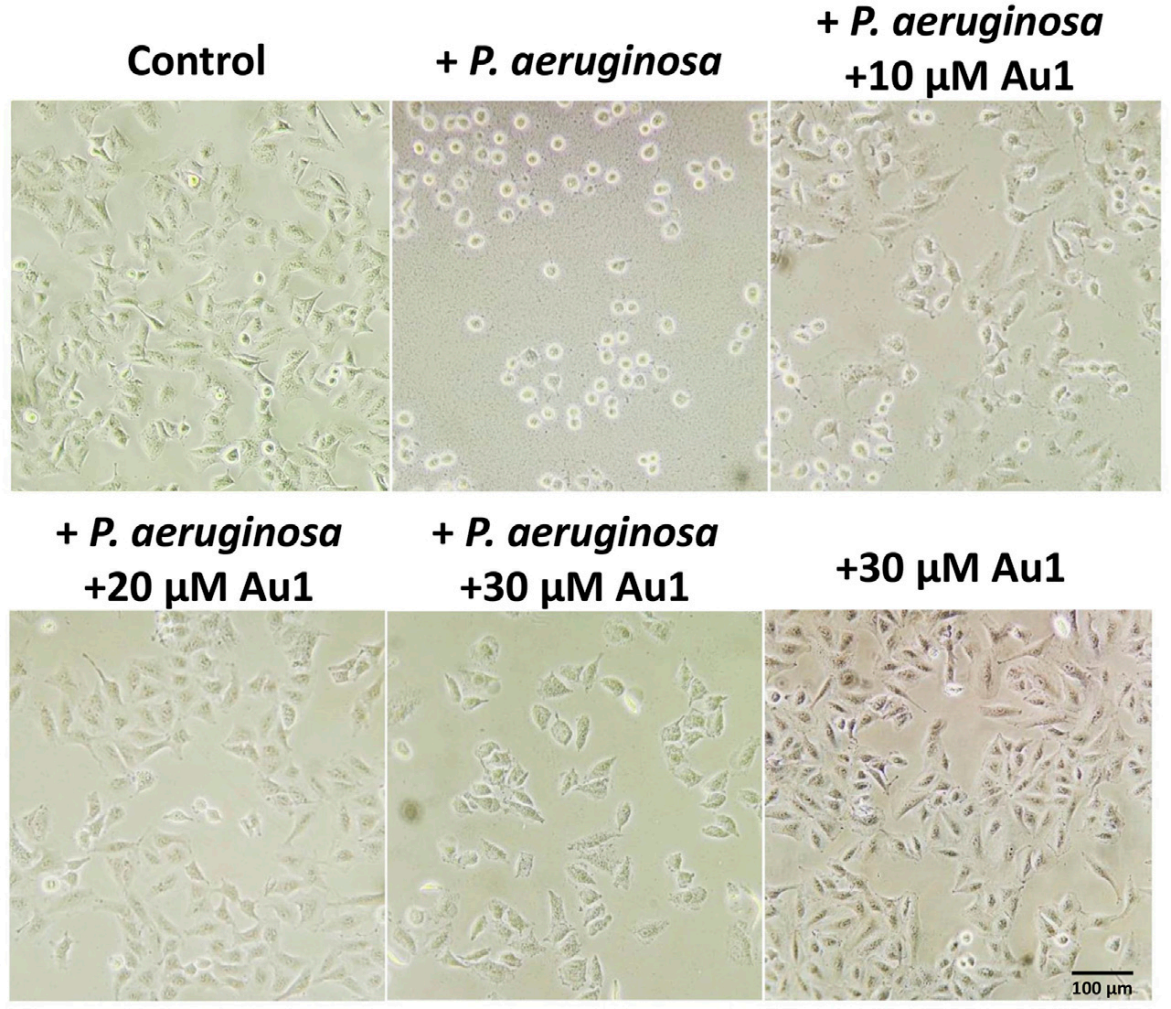
Bacterial and mammalian cell co-culture experiment. Human alveolar basal epithelial A549 cells are incubated with *P. aeruginosa* in the absence and presence of different concentrations of Au1 compound. Scale bar: 100 μm.

*P. aeruginosa* is known to cause a cytotoxic effect on eukaryotic cell via translocation of bacterial effectors into the host cells (Meirelles and Newman, 2018; Malhotra et al., 2019). To further explore the potential application of Au1 in treatment of infections caused by *P. aeruginosa*, a mammalian and bacterial cell co-culture experiment was performed to mimic the bacterial infection *in vitro*. In brief, the human alveolar basal epithelial A549 cells were co-cultured with *P. aeruginosa* PAO1 strain (the ratio of bacteria to cells is 50:1) in the absence or presence of Au1 compound. The cytotoxic effects of *P. aeruginosa* were examined based on A549 cell morphology after 2 h incubation. As shown in Figure 5, healthy A549 cell exhibited a pebble-like shape and formed well spread monolayer. Once co-cultured with *P. aeruginosa*, majority of A549 cells retracted into round shapes with drastically reduced surface area and gradually detached from the culture plate. Encouragingly, the cytotoxic effects of *P. aeruginosa* on A549 cells were substantially attenuated with the increasing concentrations of **Au1** compound. Approximately 20 μM Au1 complex was sufficient to protect A549 cells from the *P. aeruginosa* bacterial cytotoxicity. While as high as 30 μM Au1 alone had no significant effects on A549 cell morphology.

### Au1 Eradicates *P. aeruginosa* in Mouse Skin Infection Model

**FIGURE 6:**
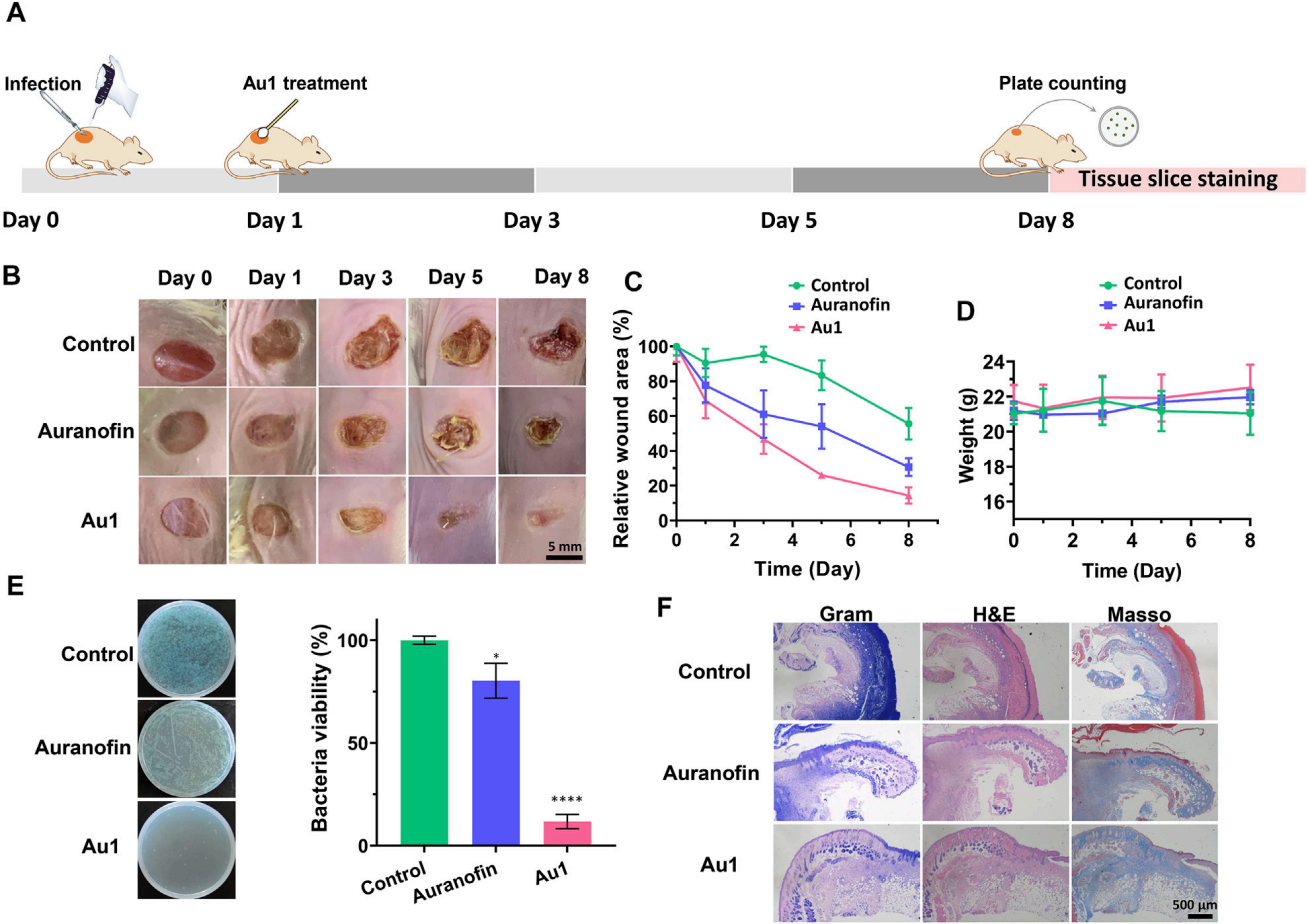
**(A)** Schematic illustration of the mice infection experiment. **(B)** The representative images and curves **(C)** of wound areas of control, aranofin-, and Au1-treated groups. Scale bar: 5 mm. **(D)** Mice weight changes within 8 days in various experimental groups. **(E)** The representative images and quantification of *P. aeruginosa* bacteria in the mice wounds. All experiments are performed in triplicated and data are presented as mean ± sd. * means *p* < 0.05, ****means *p* < 0.0001. **(F)** Gram, H&E and Masson’s trichrome staining of mice wound tissues in various experimental groups. Scale bar: 500 μm.

The above results promoted us to further investigate whether the Au1 compound was also efficacious in an *in vivo* animal infection model. To this end, we used a mouse wound infection model to assess the anti-*P. aeruginosa* efficacy of Au1 as described previously (Liao et al., 2017b). In brief, a circular wound with a diameter of 6 mm was created on the flank of mice, and approximately 5 × 10^8^ CFU of *P. aeruginosa* PAO1 were dipped onto the wound by medical cotton swab to induce infection. Treatment is administered on the 1st day with auranofin or Au1 compound (50 μM, 20 μL) was applied to the wounds. The wound sizes and mice weights were recorded each day. On the 8th day, the infection sites were excised and part of the tissues was fixed in 4% paraformaldehyde and embedded in paraffin. Four-micrometer sections were cut, stained with Gram, H&E, and Masson’s trichrome stain. The remained tissues were homogenized and serially diluted for CFU quantification (Figure 6A).

As shown in Figures 6B, C, the wound healing rate in the Au1-treated group was much faster than that in the auranofin-treated group or control group. In particular, the infected wound was almost completely recovered on the 8th day after Au1 treatment. In addition, there were no significant changes in the mice weights in all the three experimental groups, indicating that the application of Au1 or auranofin exhibited no significant toxicity to the mice (Figure 6D). The quantification of bacterial CUF in the infection sites showed that the Au1 treatment significantly reduced the live bacteria by 90% when compared to that in the control group. Whereas, approximately 70% of bacteria remained live in the auranofin-treated group (Figure 6E).

In addition, we performed histological analyses of the infected tissue to further assess the wound healing (Figure 6F). Gram staining results showed the presence of a large number of bacteria in the infected wound tissue in the control and auranofin-treated groups (blue colour indicates the presence of bacteria). In contrast, no bacteria was observed in Au1-treated wounds, indicative of the remarkable antibacterial effect of Au1. At the same time, H&E staining results showed that scabs grew in the wound of the control and auranofin-treated groups, while the wound epidermis of the Au1 group tended to be intact. Moreover, hair follicles began to appear in the wounds of the Au1 group, indicating that the wounds were healed. Masson staining results indicated the formation of blue-stained collagen fibers in different groups. The deposition of collagen fibers in the Au1 group was significantly better than that in the control and auranofin groups. The above results further indicated that Au1 had good antibacterial activity *in vivo* and promoted wound healing.

## Conclusion

In summary, we reported a gold(I) carbene compound, named as Au1, with excellent bactericidal activity against human pathogenic bacteria *P. aeruginosa. In vitro* studies showed that Au1 also has broad-spectrum antibacterial properties towards a panel of Gram-negative and -positive bacteria. Unlike antibiotics, continuous exposure of *P. aeruginosa* to Au1 compound does not induce drug resistance. At sub-lethal concentrations, Au1 substantially attenuates the virulence expression of *P. aeruginosa* and exhibits synergistic effects with antibiotics to inhibit bacterial growth. FE-SEM and AFM analysis of the *P. aeruginosa* bacteria reveals that Au1 treatment disrupts the bacterial membrane integrity, and thereby leading to significantly morphological changes of *P. aeruginosa* cell. Further RNA-seq results reveals Au1 could severely perturb several pathways in *P. aeruginosa*. In particular, the transcriptional levels of genes involved in riboflavin and trehalose biosynthesis are remarkably down-regulated by Au1. Since riboflavin derivatives are essential cofactors for a myriad of enzymes and trehalose is required for bacterial membrane stabilization, the bactericidal activity of Au1 could be partially attributed to the perturbation of the two essential bacterial metabolism pathways. Finally, we further verified that Au1 could efficiently eradicate *P. aeruginosa* in a mouse skin infection model. In a word, our studies demonstrate that the Au1 compound has great potential as a novel antibacterial agent for future clinical application to cope with current crisis of antimicrobial resistance.

## Data Availability

The original contributions presented in the study are publicly available. This data can be found here: https://www.ebi.ac.uk/ena/browser/view/, PRJEB51645.
