# Supplementary material for: Identification of an Au(I) N-Heterocyclic Carbene Compound as a Bactericidal Agent Against Pseudomonas aeruginosa
Source: Front Chem. 2022 Apr 28;10:895159. doi: 10.3389/fchem.2022.895159 (PMC9096233; doi:10.3389/fchem.2022.895159)
Supplement: Supplementary file 1 [file DataSheet1.docx]

Supplementary Material

# Supplementary Figures and Tables

## Supplementary Figures


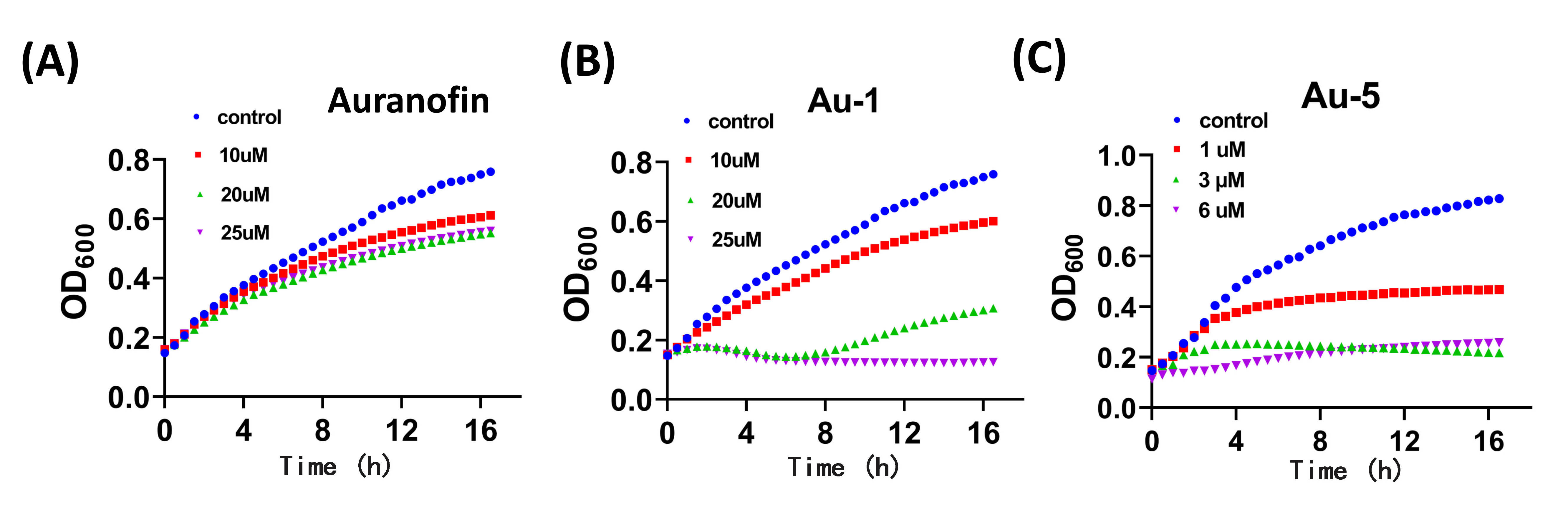


**Figure S1.** *P. aeruginosa* growth curves in the presence of different concentrations of (A) aruanofin (B) **Au-1** and (C) **Au-5**.


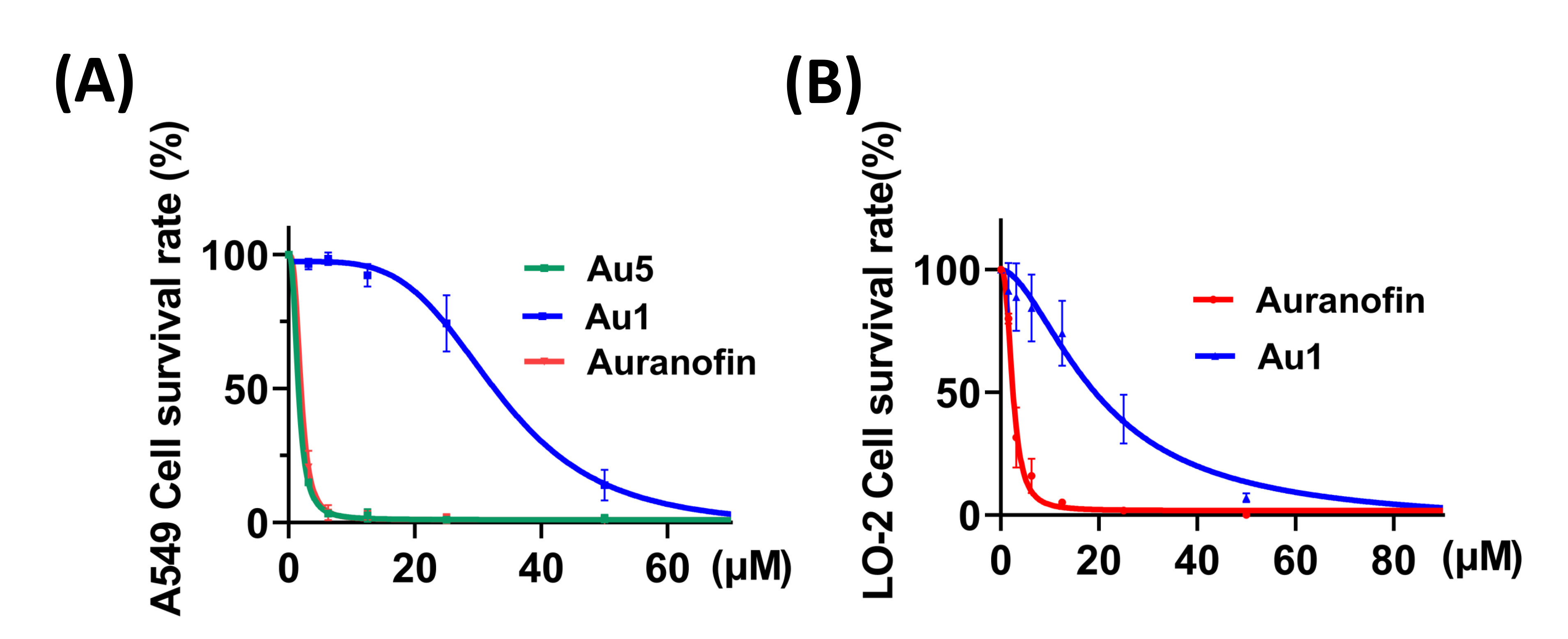


**Figure S2.** Cytotoxicity of gold(I) compounds toward (A) A549 cells and (B) LO2 cells.


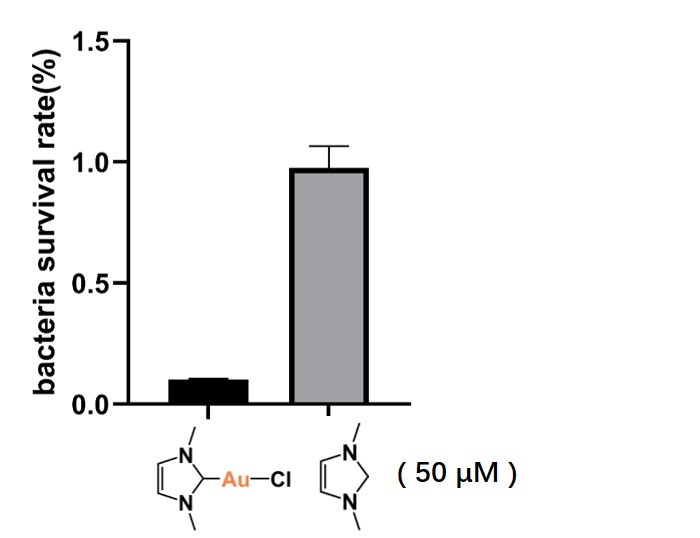


**Figure S3.** The growth of *P. aeruginosa* was not significantly affected after 16 h incubation with the cabene ligand of Au1 at 50 μM.


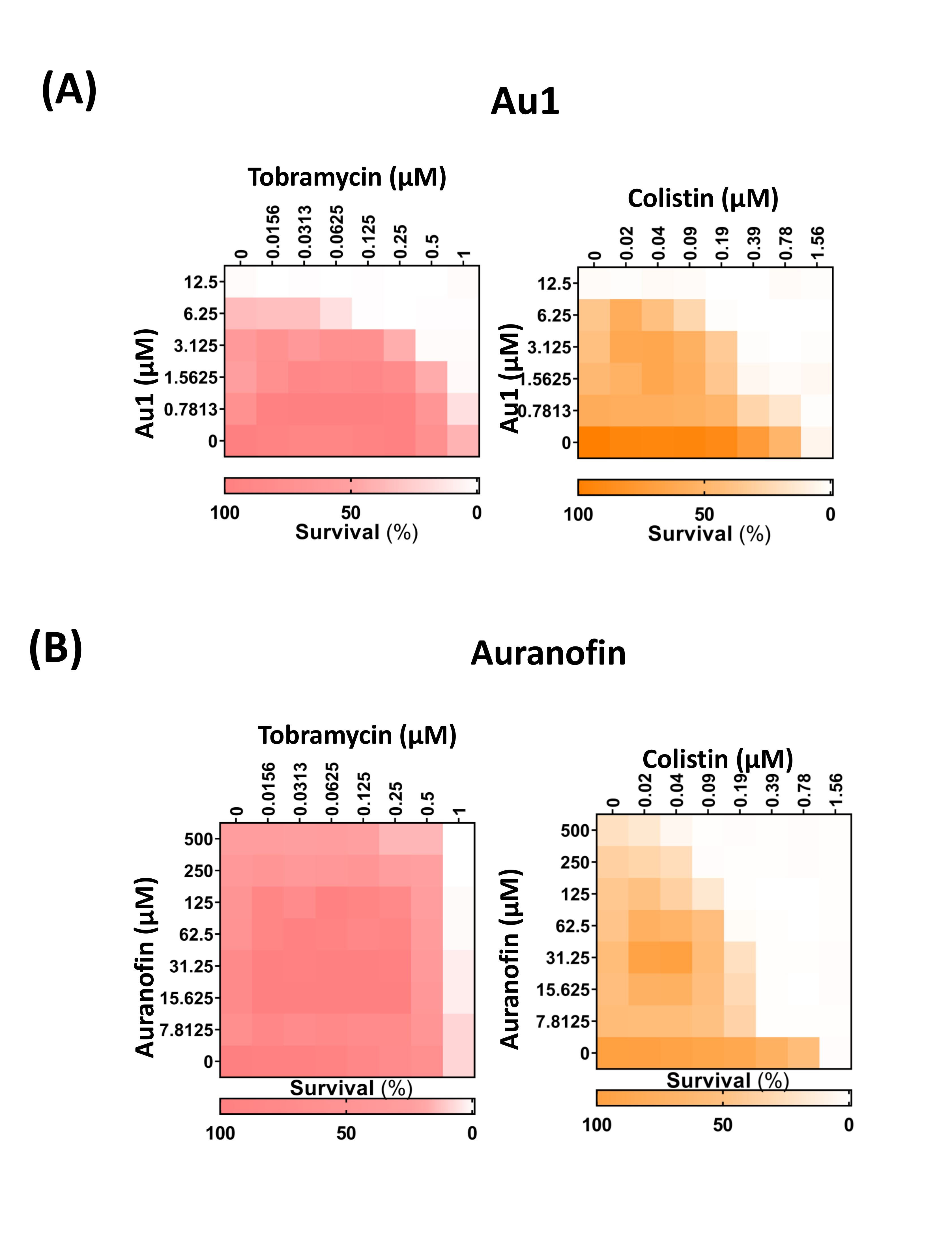


**Figure S4.** Heat plots of checkerboard assays for gold(I) compounds in combination with different antibiotics against *P. aeruginosa*. (A) Analysis of the synergistic effects between **Au1** and tobramycin (left), colistin (right). (B) Analysis of the synergistic effects between auranofin and tobramycin (left), colistin (right).


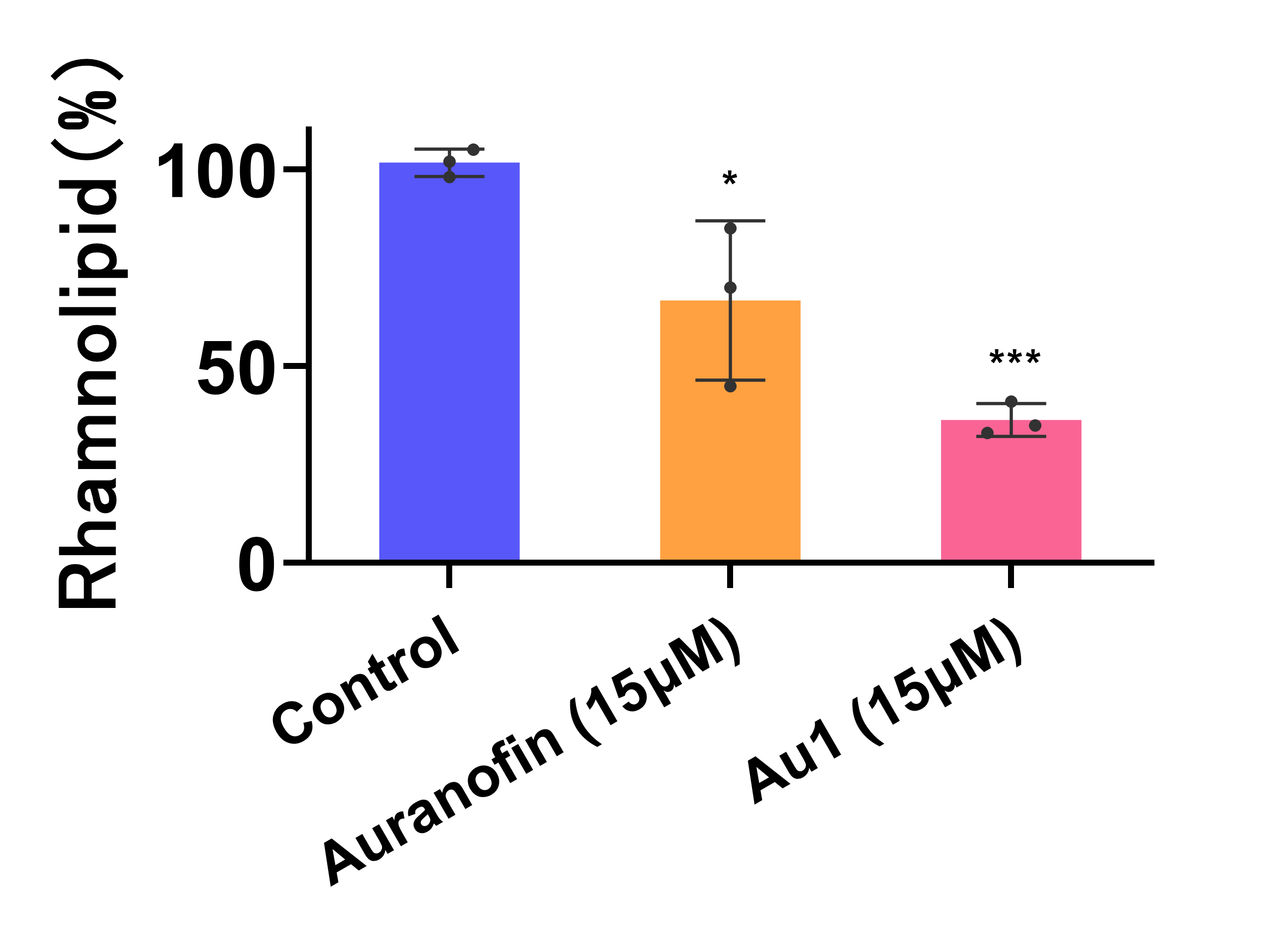


**Figure S5.** Inhibition of rhamnolipid biosynthesis in *P. aeruginosa* by 15 μM auranofin and **Au1**. The asterisks indicate significant difference from the control group (*, 0.01 < p < 0.05 ***, 0.0001 < p < 0.001)


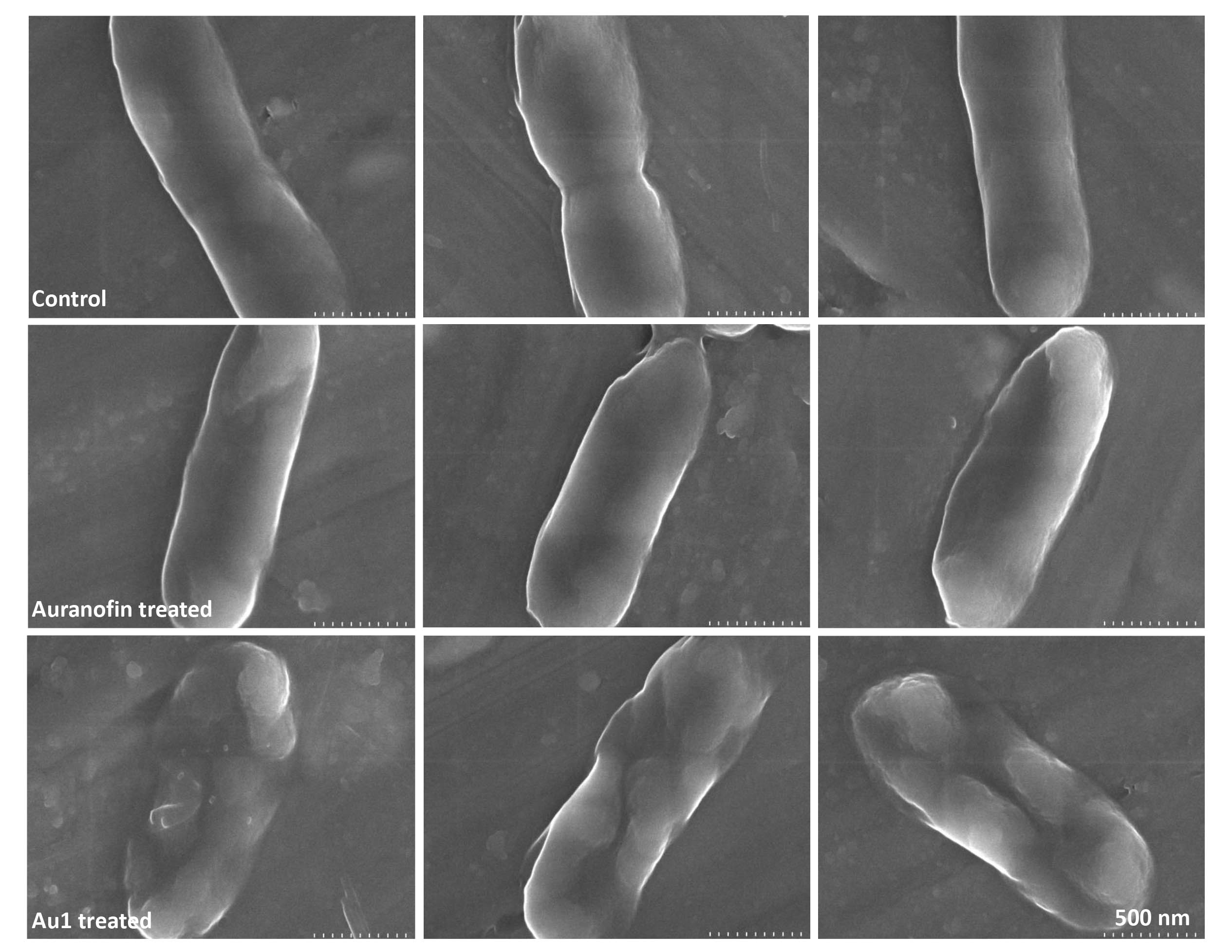


**Figure S6.** FE-SEM images of *P. aeruginosa* cells after treatment of auranofin and **Au1**. The data reveals that **Au1** significantly damages the bacterial membrane. Scale bar: 500nm.


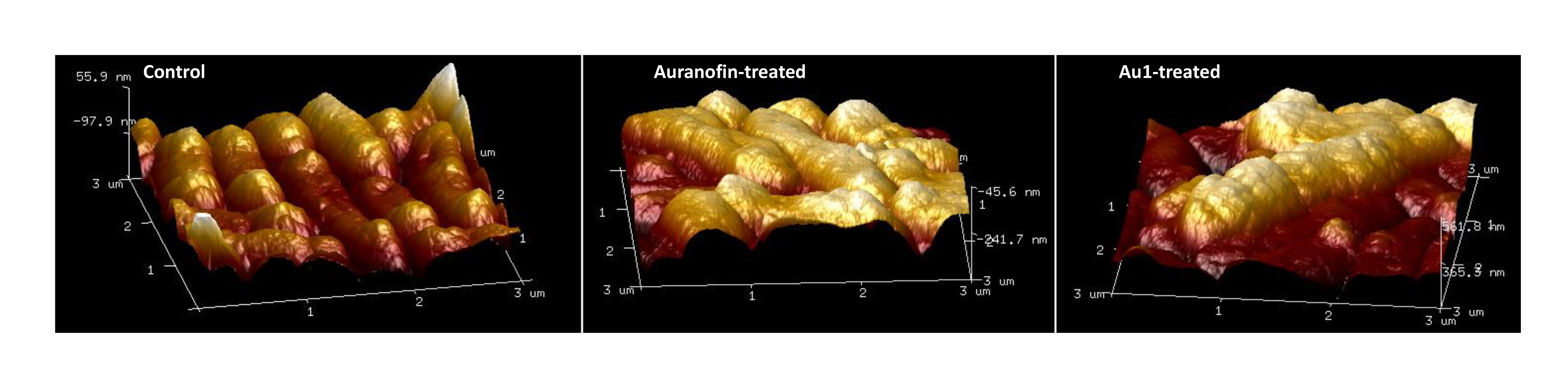


**Figure S7.** AFM images of *P. aeruginosa* cell surface after auranofin and **Au1** treatment. The bacterial cell surface in **Au1**-treament group exhibits significantly enhanced roughness.


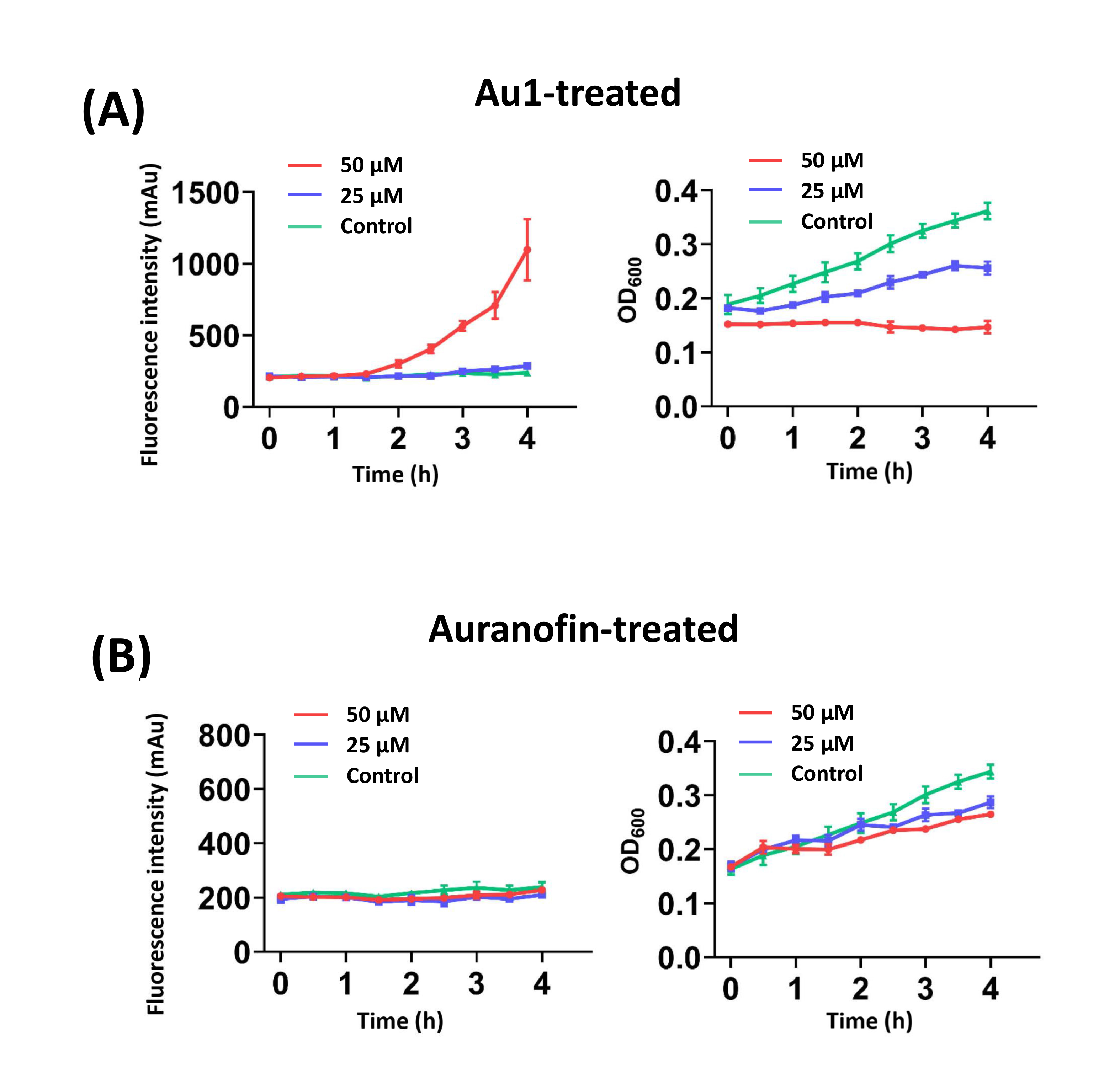


**Figure S8.** Kinetic analysis of the *P. aeruginosa* bacterial membrane permeation caused by (A) **Au1** and (B) auranofin. The red fluorescence signals (excitation at 525 nm/emission at 610nm) of bacteria are normalized to the OD_600_ values in the course of time.


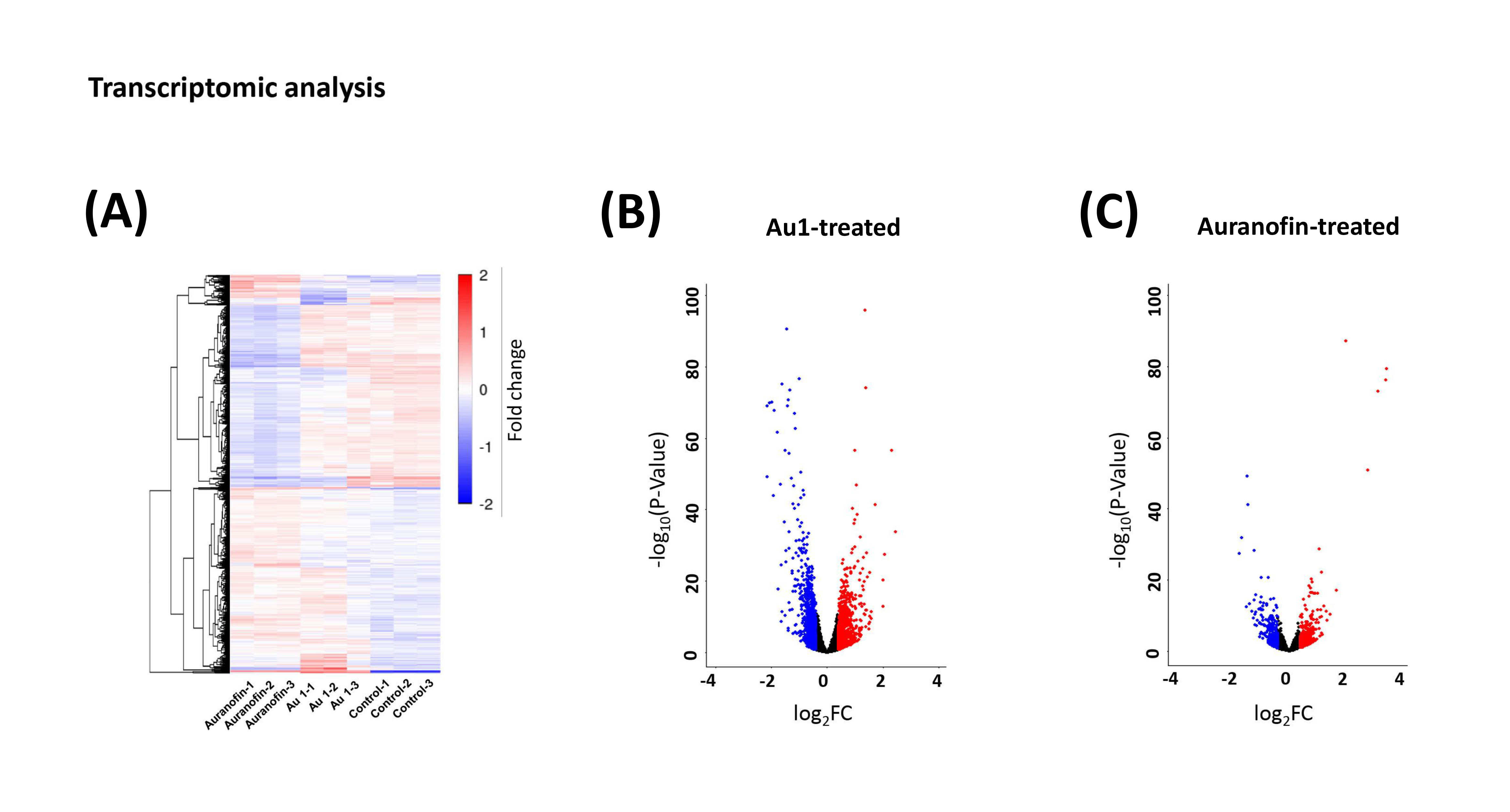


**Figure S9.** (A) Gene transcription profiles from *P. aeruginosa* treated with 15 μM **Au1** and auranofin for 4 h. Volcano plots showing the altered genes in the (B) **Au1** or (C) auranofin treated groups compared to the control group.


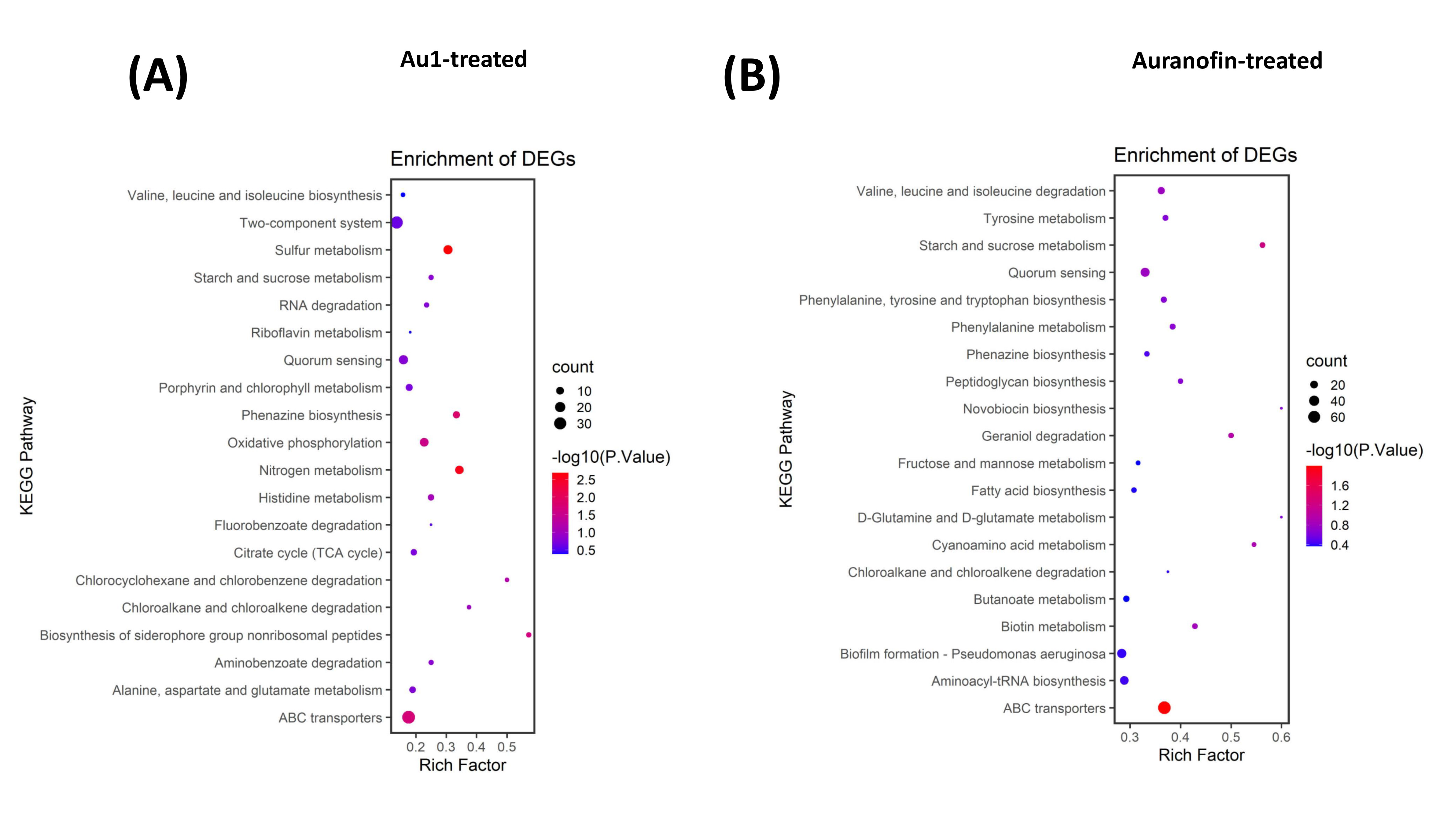


**Figure S10.** KEGG pathway enrichment analysis of the significantly altered genes in *P. aeruginosa* after **Au1** (A) and auranofin (B) treatment.


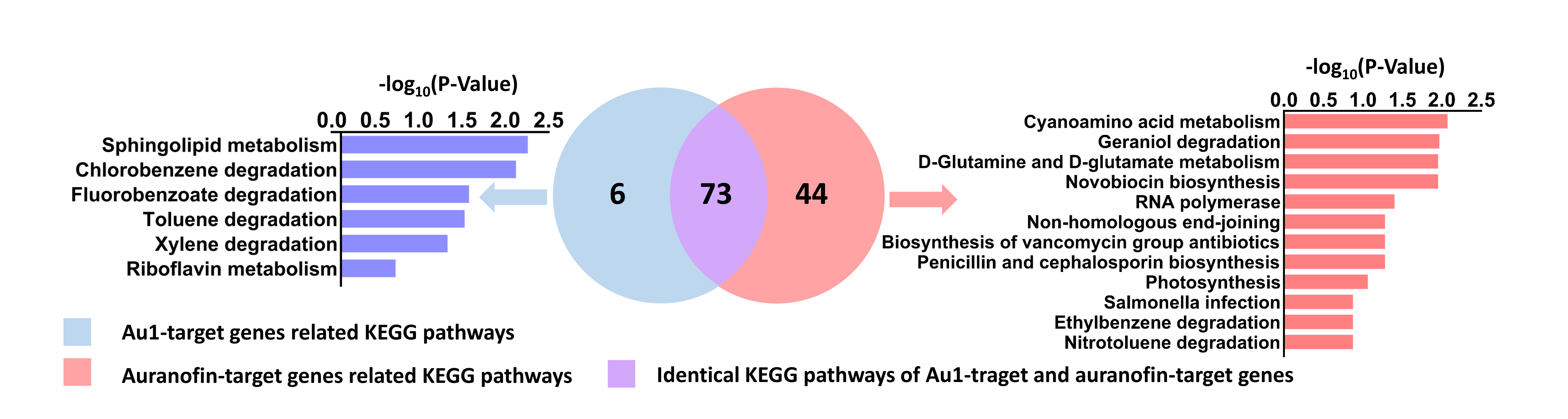


**Figure S11.** Analysis of altered genes related KEGG pathways in **Au1**- and auranofin-treated groups.

## Supplementary Table

**Table S1.** The minimal inhibitory concentrations (MIC) of auranofin, **Au1** and antibiotics towards various bacterial species

| Compound | Auranofin  (μM) | **Au1**  (μM) | Ciprofloxacin  (μM) | Tobramycin  (uM) | Polymyxin  (uM) | Carbenicillin  (uM) |
| --- | --- | --- | --- | --- | --- | --- |
| *Pseudomonas aeruginosa* PAO1 | >100 | 25 | <1.56 | 12.5 | <1.56 | >100 |
| *Staphylococcus aureus* *Newman* | <1.56 | <1.56 | <1.56 | 25 | >100 | 6.25 |
| *Burkholderia cepacia* *J2315* | >100 | 25 | 25 | >100 | >100 | >100 |
| *Escherichia* *coli MG16552* | >100 | <1.56 | <1.56 | 50 | <1.56 | 25 |
